# National community pharmacy NHS influenza vaccination service in Wales: a primary care mixed methods study

**DOI:** 10.3399/bjgp16X684349

**Published:** 2016-03-11

**Authors:** Andrew M Evans, Fiona C Wood, Ben Carter

**Affiliations:** Public Health Wales NHS Trust, Temple of Peace and Health, Cardiff.; Institute of Primary Care & Public Health, Cardiff University School of Medicine, Cardiff.; Institute of Primary Care & Public Health, Cardiff University School of Medicine, Cardiff.

**Keywords:** community pharmacy services, general practice, immunisation, pharmacy

## Abstract

**Background:**

Influenza is a significant cause of morbidity and excess mortality, yet vaccine coverage in the UK remains below target. Community pharmacies are increasingly being promoted as an alternative to vaccination by GPs.

**Aim:**

To explore and verify the factors that influence the relative performance of pharmacies providing NHS influenza vaccinations.

**Design and setting:**

A mixed methods study utilising qualitative, semi-structured interviews and quantitative analysis of predictors of vaccination numbers in community pharmacies in Wales.

**Method:**

Interviews were conducted with 16 pharmacists who participated in the Welsh national pharmacy influenza service in 2013–2014. A purposive sampling strategy was used. Qualitative findings were analysed using framework analysis. Potential predictors of vaccination numbers were identified from interviews and a literature review, and included in a multivariable regression model.

**Results:**

The contribution of community pharmacies towards vaccination in Wales is small. Findings suggest that community pharmacies reach younger at-risk individuals, in whom vaccine uptake is low, in greater proportion than influenza vaccination programmes as a whole. Extended opening hours and urban locations were positively associated with the number of vaccinations given, although pharmacists reported that workload, vaccine costs, unforeseen delays, lack of public awareness, and GPs’ views of the service limited their contribution. Pharmacists, aware of the potential for conflict with GPs, moderated their behaviour to mitigate such risk.

**Conclusion:**

Before community pharmacies take greater responsibility for delivering healthcare services, obstacles including increasing pharmacist capacity, vaccine procurement, health service delays, managing GP–pharmacy relationships, and improving public awareness must be overcome.

## INTRODUCTION

Influenza is a significant cause of morbidity and excess winter mortality in the UK.^1^,^2^ Those at higher risk, including people with underlying health conditions such as cardiac or respiratory disease, and those aged ≥65 years, are eligible for immunisation, the cost of which is met by the NHS.^3^ The World Health Organization recommends that at least 75% of people ≥65 years should be vaccinated,^4^ yet UK vaccination rates are lower than this.^3^ Strategies to increase vaccination have included encouraging general practices to increase access, and promoting early morning, evening, and weekend appointments.^5^ More recently, community pharmacists have been used as an alternative provider of influenza vaccination and this has been found to increase immunisation rates and meet service user needs.^6^–^8^

Community pharmacy-based vaccination is not a new phenomenon. In the US pharmacists have been giving influenza vaccinations since the mid-1990s.^9^,^10^ In the UK a pilot was undertaken as early as 2002,^11^ and non-NHS influenza vaccination services are widely available from community pharmacies.^12^ There is evidence that vaccinations are given safely by pharmacists,^13^,^14^ and that access to pharmacies is greatest in areas of social deprivation,^15^ in which influenza vaccination rates are low.^16^

Influenza vaccination is an example of how pharmacies could contribute to improving public health and widening access. Pharmacy bodies in England have called for community pharmacies to take on aspects of GP workload as a potential solution to the workload pressures faced by GPs,^17^ and this view has been supported by the British Medical Association.^18^ The extent to which community pharmacies deliver influenza vaccination could be indicative of the sector’s capacity to make a significant contribution given previous concerns about their capacity to do so.^12^ However, other factors may influence the participation of individual pharmacists in extended roles; previous studies have shown that shortages of time, disruption to dispensing, vaccine availability, and lack of GP support are barriers to pharmacist involvement in vaccination programmes.^12^,^19^–^27^ The applicability of these studies is limited, however, as most were conducted outside the UK.

In July 2015, NHS England announced its intention to commission a national NHS influenza vaccination service from community pharmacies. The move has been controversial and, although it has been welcomed by pharmacy representatives,^28^ it has been met with resistance from those representing GPs. The latter have expressed concern about the adverse financial impact that pharmacy provision could have on GP practices.^29^,^30^ Before 2015, Wales was the only part of the UK to have a national pharmacy influenza service. This study provides an insight into the challenges that must be met for community pharmacies to take an expanded role in influenza vaccination and other primary care services.

**How this fits in**

The recent announcement from NHS England that all pharmacies could provide NHS influenza vaccinations from 2015 has been met with resistance from GP representatives. Factors that influence the number of NHS influenza vaccinations given by pharmacies in the UK have not been evaluated. This study identified that extended opening hours and urban locations are positive predictors of vaccination numbers. Workload, vaccine costs, delays outside the pharmacists’ control, lack of public awareness, and GPs’ views of the service limited their contribution, however.

## METHOD

A mixed methods study was conducted in Wales during 2014. The mixed methods approach was chosen to allow both the pattern of pharmacy vaccination and the reasons underlying that pattern to be explored. The qualitative phase explored pharmacists’ experiences of providing the service, including their views on facilitators and barriers, through telephone interviews. The findings of the qualitative phase informed the quantitative phase, which used data on NHS vaccinations obtained for all community pharmacies providing the pharmacy influenza vaccination service (‘the service’) in Wales between 1 October 2013 and 31 March 2014. Data were provided by the NHS Wales Shared Services Partnership and included the number of vaccinations given, and age, sex, and self-reported eligibility of each person vaccinated. From pharmacist interviews and the literature, key factors that may explain the number of vaccinations given by pharmacies were explored. Potential predictors were found to be deprivation; workload; opening hours (that is, evening and weekend opening); ownership (that is, multiple or independent); and whether the pharmacy served a rural or urban area (Appendix 1).

The quantitative phase involved descriptive analysis of pharmacy activity data. The number of vaccinations given by pharmacies was transformed to the natural logarithmic scale and the relationship between the number of vaccinations and potential predictors was analysed by *t*-test for binary, and univariable linear regression for continuous variables. Explanatory variables and potential confounders found to explain vaccination numbers were subsequently included in a multivariable linear model. Analyses were carried out using Stata (version 13.1).

In the qualitative phase, the sampling frame included pharmacists if they practised at a pharmacy providing the service in the 2013–2014 influenza season and provided at least one vaccination during that period. Pharmacists were identified by categorising pharmacies into performance quintiles on the basis of the number of vaccinations given. Pharmacies in the first (that is, providing the highest number of vaccines) and fourth quintiles were selected for detailed investigation, increasing the likelihood of identifying differing themes. Pharmacies in the fifth quintile were not selected because they gave too few vaccinations to have been able to form a sufficiently rounded view of all aspects of the service and its barriers and facilitators. From an initial list of 78, a purposive sample of 43 pharmacies was compiled with a balance of geographical distribution, ownership, dispensing volumes, and the proportion of vaccinations given to people not vaccinated in the previous influenza season. Pharmacists giving vaccinations at each of the pharmacies in the sample frame were identified by cross-referencing claims data with the online register of UK pharmacists maintained by the General Pharmaceutical Council (*n* = 44). A topic guide was devised from the literature review; interviews were audiotaped and transcribed. The resulting data were analysed using framework analysis.^31^ Transcripts were read, re-read, and annotated to identify the main themes and sub-themes. Annotations were coded with emerging codes continuously compared between transcripts. Data analysis was conducted by the principal investigator and overseen by the co-authors.

## RESULTS

### Number of vaccinations provided by pharmacies

A total of 7861 vaccinations were given from 195 pharmacies (27.3% of the total number of pharmacies in Wales, *n* = 714) during the study period. The characteristics of pharmacies are shown in Table 1. The mean number of vaccinations per participating pharmacy was 40.31 (SD 46.95; *n* = 195). More than 100 vaccinations were given at 10.8% of pharmacies (21 out of 195); the highest number of vaccinations given at a single pharmacy was 282. Fifty-six pharmacies (28.7%) provided fewer than 10 vaccinations.

**Table 1.** Characteristics of pharmacies participating in NHS seasonal influenza vaccination service 2013–2014

	**All pharmacies, *n* (%)**	**Participating pharmacies, *n* (%)**	**Non-participating pharmacies, *n* (%)**
**Pharmacy type**			
Independent	240 (33.61)	47 (24.10)	193 (37.19)
Multiple	474 (66.39)	148 (75.90)	326 (62.81)

**Rural Urban Classification**			
Urban	507 (71.01)	136 (69.74)	371 (70.13)
Rural	199 (27.87)	56 (28.71)	153 (28.92)
Not available	8 (1.12)	3 (1.54)	5 (0.95)

**Deprivation quintile**			
1 (lowest)	94 (13.17)	22 (11.28)	72 (13.87)
2	96 (13.45)	28 (14.36)	68 (13.10)
3	174 (24.37)	52 (26.67)	122 (23.51)
4	180 (25.21)	54 (27.69)	126 (24.28)
5 (highest)	170 (23.81)	39 (20.00)	131 (25.24)

**Extended-hours pharmacy**			
Yes	37 (5.18)	24 (12.31)	13 (2.50)
No	677 (94.82)	171 (87.69)	506 (97.50)

**Average monthly prescription volume quintile**			
1 (lowest)	143 (20.03)	37 (18.97)	106 (20.42)
2	143 (20.03)	39 (20.00)	104 (20.04)
3	143 (20.03)	36 (18.46)	107 (20.62)
4	143 (20.03)	46 (23.59)	97 (18.69)
5 (highest)	142 (19.89)	37 (18.97)	105 (20.23)

Total	714	195	519

The total number of people receiving NHS influenza vaccination in Wales in 2013–2014 was 668 780.^32^ The 7861 individuals vaccinated in pharmacies represent only 1.18% of all those vaccinated. The mean age of individuals vaccinated in pharmacies (*n* = 7861) was 60.3 years (95% CI = 59.9 to 60.6 years, range 18–100 years). Data on sex were available for 7854 claims (99.9%). Most vaccines were given to females (4533, 57.7% versus 3321, 42.3%).

The most common eligibility criterion was being aged ≥65 years (4081, 51.9%, Table 2).

**Table 2.** Number of NHS seasonal influenza vaccinations provided by community pharmacies in Wales by NHS eligibility criteria 2013–2014 (*n* = 7861)

**Eligibility criteria**	***n* (%)**
Aged ≥65 years	4081 (51.9)
Chronic respiratory disease^a^	1564 (19.9)
Diabetes^a^	639 (8.1)
Carer	571 (7.3)
Chronic heart disease^a^	280 (3.6)
Pregnancy	233 (3.0)
Immunosuppressed^a^	174 (2.2)
Chronic neurological disease^a^	95 (1.2)
Other (as specified in PGD)	76 (1.0)
Household contact of immunocompromised individuals	40 (0.5)
Chronic kidney disease^a^	38 (0.5)
Designated first aider	34 (0.4)
Chronic liver disease^a^	20 (0.3)
People living in long-stay residential care homes or other long-stay care facilities	10 (0.1)
Community first responder	6 (0.1)

Total	7861

a Clinical risk groups in those aged <65 years. PGD = Patient Group Direction.

The proportion of people aged <65 years and in a clinical risk group vaccinated was higher in pharmacies than the proportion across the vaccination programme as a whole (pharmacies 2812, 35.8%; programme 163 377, 24.4%, mean difference 11.3%, 95% CI = 10.3% to 12.4%, *P*<0.001).^33^

Reported vaccination histories were available for all 7861 individuals vaccinated: 1960 individuals (24.9%) reported having not been vaccinated in 2012–2013, a further 5035 (64.1%) were vaccinated by their GP, and 485 (6.2%) received an NHS pharmacy vaccination, with the remaining 381 individuals (4.8%) vaccinated at a range of locations including vaccination provided by their employer and paid for vaccination at a community pharmacy.

### Predictors of vaccination numbers

The results of the univariable analyses are shown in Tables 3 and 4. After including statistically significant predictors in a multivariable model there was some evidence of a difference in the mean number of vaccinations, although neither having an urban location (regression coefficient 0.37, 95% CI = −0.05 to 0.78, *P* = 0.08) nor having extended opening hours (regression coefficient 0.55, 95% CI = −0.03 to 1.12, *P* = 0.06) reached statistical significance at the 95% confidence level.

**Table 3.** Association between (continuous variable) predictors and the number of NHS influenza vaccinations

**Predictor**	**β coefficient**	***P*-value**	**95% CI**
Deprivation score	0.010	0.893	−0.131 to 0.015
Average monthly prescription volume	0.020	0.351	−0.022 to 0.062

**Table 4.** Association between (binary variable) predictors and the number of NHS influenza vaccinations

**Predictor**	***n***	**Mean difference in number of vaccinations**	**95% CI**	***P*-value**
**Rural Urban Classification**				
Rural location	56	–	–	–
Urban location	136	8.385	−1.712 to 18.260	0.032

**Trading hours**				
Normal	171	–	–	–
Extended	24	15.796	−2.343 to 42.964	0.031

**Pharmacy ownership**				
Multiple	148	–	–	–
Independent	47	6.506	−5.750 to 21.420	0.175

### Pharmacist interviews

In total 44 pharmacists were invited to participate in the study. Sixteen responded, giving a response rate of 36.4%. The characteristics of participants are shown in Table 5. Data saturation was assessed after 15 interviews, and recruitment was continued to 16 interviews to ensure the number of interviews with pharmacists from the high- and low-giving quintiles was equal.

**Table 5.** Characteristics of interview participants

**Participant number**	**Sex**	**Years in pharmacy practice**	**Postgraduate qualifications**	**Position in pharmacy**	**Pharmacy type**	**Full-/part-time working**
H1	M	16–20	None	Owner	Ind	Full
H2	M	6–10	Certificate	Employee	Ind	Full
H3	F	16–20	PhD	Manager	Mt	Part
H4	M	1–5	None	Manager	Mt	Full
H5	F	11–15	Masters	Manager	Ind	Part
H6	F	11–15	Diploma	Employee	Mt	Full
H7	F	≥31	None	Manager	Mt	Full
H8	M	6–10	Diploma	Employee	Mt	Full
L1	M	11–15	Diploma	Owner	Ind	Full
L2	M	26–30	Certificate	Owner	Ind	Full
L3	F	26–30	None	Employee	Mt	Part
L4	M	21–25	Masters	Owner	Ind	Full
L5	F	6–10	Diploma	Manager	Mt	Full
L6	F	≥31	None	Manager	Mt	Full
L7	M	26–30	None	Owner	Ind	Full
L8	F	6–10	None	Manager	Mt	Part

F = female. H = provided high number of vaccinations. Ind = independent. L = provided low number of vaccinations. M = male. Mt = multiple.

Interviews identified three main themes: pharmacy factors; public awareness; and external factors. These with the sub-themes and codes emerging under each are detailed in Table 6. The main themes, and most significant sub-themes, are discussed in a linear fashion; however, many are intrinsically linked. The perceptions of pharmacists giving comparatively high and low numbers of vaccinations are discussed together using illustrative quotes with participants identified by the codes H and L, respectively.

**Table 6.** Themes, sub-themes, and codes describing pharmacists’ views of the determinants of the number of NHS influenza vaccinations provided at pharmacies

**Main theme**	**Sub-themes**	**Codes**
**Pharmacy factors**	Pharmacist numbers	Other pharmacist(s) available to maintain day-to-day functions of the pharmacy
		Maximising the number of pharmacists able to immunise
		Reliance on pharmacist to provide the (flu) service

	Extended trading	Evening opening hours
		Weekend opening hours

	Pharmacy location	Proximity/co-location with GP practice
		Footfall
		Pharmacy as part of defined community
		Rurality
		Town centre
		Population served by pharmacy

	Staff support	Delegation of roles to staff
		Training
		Recruiting patients

	Flexibility to offer vaccinations	Drop in/no appointments needed

	Identifying patients	Checking against prescriptions
		Promoting vaccination to people accessing other pharmacy services
		Targeting specific at-risk groups

	Planning approach	Business focus — profitability
		Providing a high-quality professional service (altruism)
		Capacity or absence of planning
		Planning to supplement (mop up) GPs

	Impact on other services	Reducing level of other services to accommodate vaccination

	Premises/facilities	Number of consultation rooms

**Public awareness**	Word of mouth	Snowballing
		Staff raising awareness

	Promotional material	Corporate display materials
		Insufficient/inadequate/delayed promotional materials
		Promotional displays
		Restrictions on advertising
		No awareness of NHS service
		Decision not to promote by pharmacy
		Importance of flu vaccination/eligibility

**External factors**	Finance	Profit motive
		Incentives
		Unimportance of profit

	GP relationships	Reciprocity
		Conflict avoidance
		Views of GPs and practice staff

	Vaccine availability	Supply chain shortages
		Purchasing restrictions
		Uncertain demand

	Administrative burden	Paperwork

	Commissioning processes	Accreditation processes
		Patient Group Direction
		Approval by health board

#### Pharmacy factors

How does pharmacy workload affect participation? Half of the pharmacists (*n* = 8) reported that workload was an important determinant of the number of vaccinations. In particular, having more than one pharmacist present helped by preventing disruption to the other activities:

‘... we’ve got two pharmacists here so it means that dispensing continues without disrupting the normal day-to-day activities.’

(H5)

Some (*n* = 8) reported prioritising vaccination over other services:

*‘I did do less MURs* [Medicine Use Reviews]*, part of that because I was doing vaccinations and had the other services to deal with.’*

(L5)

One pharmacist gave an insight into pharmacies’ capacity to provide the service:

‘... there’s just not enough pharmacist hours in branch to do everything that pharmacists need to do … because we are busy you kind of don’t drive it as much as you could.’

(L5)

How do a pharmacy’s opening hours affect provision? A few pharmacists who provided a comparatively high number of vaccinations considered the availability of vaccination at weekends, at lunchtime, and in the evening specifically to be important determinants of vaccination numbers:

‘They also liked our opening times as well. They liked that you could come in in the evening and have it done.’

(H6)

‘I’ve got a friend who’s an electrician he’s working till half five at night goes out to work at half eight in the morning. He came to see me on a Saturday afternoon and had his flu vaccination.’

(H1)

This was corroborated by comments from pharmacists who gave low numbers of vaccines:

‘We don’t open on a Sunday … we close at lunchtime.’

(L6)

Pharmacists reported taking different approaches to delivery of the service and their role to support increasing vaccine uptake. These approaches can be split into two broad categories. The first, predominant among pharmacists giving comparatively high numbers of vaccinations, was to adopt a structured approach. This included ensuring that the pharmacy had facilities and processes that supported service delivery, improving efficiency, and maximising capacity to offer the service:

‘I think it was because I had everything to hand, everything laid out, I had all the paperwork. I planned the paperwork so I got each person that’s going to present I’ve already prepped 10 or 20 forms ready for them to turn up on a rolling basis.’

(H5)

‘We booked appointments to make sure we did have staff in that could deliver the vaccinations.’

(H8)

There are potentially many reasons why a pharmacy would adopt this approach. One pharmacist explained:

‘Every service we do we are quite keen that it’s gotta be profitable, there’s no point, we don’t see the point of doing something that doesn’t benefit profitability in any way.’

(H2)

Most pharmacists (*n* = 5) adopting a structured approach also implied that they were more responsive to people’s demands than were GP surgeries:

‘... you have to book an appointment at the surgery, there are only certain surgery times … so the patients liked it in terms of they could just walk in and have it straight away.’

(H6)

The second approach could be described as altruistic and was prevalent among pharmacists providing comparatively low numbers of vaccinations, who emphasised that providing vaccinations was, for the pharmacy profession in the UK, a recent development. These pharmacists took what they saw as a more public service (and ultimately conservative) approach.

Some of these pharmacists (*n* = 3) described offering a ‘mop up’ service, referring to only vaccinating people who had difficulty getting to their GP. These pharmacists deferred to GPs as the predominant provider:

*‘We were always going to be a support service to them* [GPs]*, for those people that couldn’t get to them for whatever reason.’*

(L4)

They also avoided criticising practices’ vaccination arrangements and reported taking steps to minimise and avoid conflict with local GPs:

‘We could have more proactively promoted it but didn’t particularly want to step on the GPs’ toes.’

(L3)

Taking a more assertive approach was considered a risky strategy that would damage relationships with GPs, with a more substantial adverse impact on business in the longer term:

‘If you try and push too much it does get their back up a bit and when you’ve got good relationships, I know some of the big companies are really hammering it and what you gain there you lose in a lot of other things.’

(L8)

Only two pharmacists, however, reported that providing the service had had a detrimental impact on relationships with GPs:

*‘...* [the practice manager] *was very much concerned with the fact that we were solely there to try and mop up.’*

(L1)

*‘Once they saw a lot of patients choosing to come here* [to the pharmacy] *straight away they got really, really defensive and made all kinds of sort of threats to try and stop me from doing it.’*

(H4)

Positively, the second of these pharmacists reported that the local health board (LHB) provided mediation and that he had been able to work more closely with GPs afterwards:

‘The LHB sort of intervened and told the GP you can’t do that and suggested maybe they would provide us with a list of eligible patients that didn’t get it last year, which they did and that worked quite well. We were able to target those patients.’

(H4)

Some pharmacists (*n* = 4) reported that GPs had responded positively to the pharmacy providing the service, even encouraging people to use it:

*‘The biggest surgery locally were directing people if they missed the GPs’ appointment, GPs’ days for doing vaccinations, were saying oh* [pharmacist’s name] *is doing it over in the chemist. If you go over there she might be able to do them for you.’*

(H5)

A high number of pharmacists (*n* = 7) reported being unaware of strong views, either positive or negative, from GPs.

Over half of pharmacists (*n* = 9) thought that public awareness of pharmacy influenza vaccination was poor:

‘People didn’t know about the service.’

(L5)

‘… not terrific awareness for the NHS jabs, I think that missed out almost entirely. I’m struggling to think of people who came in and said I’ve heard you do NHS flu jabs, can I have one?’

(H7)

‘When we talk to people about it they are not always aware that we can do it. Stemming from that maybe an increased awareness would mean more people getting vaccinated.’

(H8)

#### External factors

Most pharmacists (*n* = 10) commented on the impact of various procedural delays. In particular, they reported that there had been a delay in receiving the Patient Group Direction (PGD), which provided the necessary authority to give vaccinations:

‘... we didn’t actually get the PGD until the week the service started.’

(L6)

‘... it was a very slow burner to start off but it was because the paperwork and all the sign-off for the PGD didn’t come through until after all of our local surgeries had already run their flu clinics.’

(L5)

Some pharmacists (*n* = 4), particularly those providing comparatively low numbers, thought preparation for the service had been poor, specifically identifying LHB disorganisation as a problem:

*‘They* [the LHB] *don’t strike me as particularly organised. Stuff needs to get to us on time, well in advance really not just scraping through.’*

(L7)

‘Everything came through from the Health Board fairly late on to be honest.’

(H7)

Of the pharmacists interviewed who had given a comparatively low number, half (*n* = 4) reported difficulties with securing adequate supplies of vaccine:

‘I kept on reordering and reordering. Eventually they just stopped coming in and then there was a shortage.’

(L5)

‘I’d say oh I’ve run out but I should have some in tomorrow you can try ringing about, and they never used to come back then … we’d have done more if we’d had the reliability of the supply chain.’

(L5)

Others (*n* = 2) reported that, although they were able to obtain vaccines, the costs of doing so resulted in a financial loss for the pharmacy. The result of this was that they faced a choice between meeting either their professional or commercial obligations. This was a scenario in which there was no satisfactory resolution:

*‘I had to buy them* [vaccines] *in tens which meant that three of them were wasted. So I don’t know that I made any money at all last year but I’m not completely interested in that I’m also happy to provide a service … what do they* [the LHB] *want me to think when I open the new box, think I’m never gonna get rid of these 10* [vaccines]*, turn them* [members of the public] *all away … They’re risking us, risking us turning three or four people away.’*

(L2)

## DISCUSSION

### Summary

This study highlights the complexity of delivering extended primary care services consistently through community pharmacies. The contribution of community pharmacies towards vaccination in Wales is small. Although some pharmacies demonstrated that they could provide a comparatively high number of vaccinations, most provide comparatively few. Positive findings suggest that community pharmacies reach younger at-risk individuals, in whom vaccine uptake is low, in greater proportion than influenza vaccination programmes as a whole. Extended opening hours and urban locations were positively associated with the number of vaccinations given.

### Strengths and limitations

This is the first study that has examined both quantitatively and qualitatively the association between predictors and the number of pharmacy vaccinations, taking into account all pharmacy types, locations, opening hours, and workload. It is also the first study designed specifically to assess determinants of influenza vaccine provision by pharmacists in the UK.

A limitation is perhaps the measurement of the performance of pharmacies in numbers of vaccines given, which assumes that all pharmacies serve a broadly similar at-risk population. Participants were volunteers who may have overstated their enthusiasm or how well the service was received by patients, or understated GP resistance, particularly if they believed this was critical to ensuring that they were commissioned in the future. All participants were providing NHS influenza vaccinations and findings cannot necessarily be applied to all pharmacies.

### Comparison with existing literature

Extended opening hours and an urban location were positively associated with the number of vaccinations given, supporting previous qualitative studies;^6^,^11^,^34^ however, the effect size was small and, after adjusting for confounding, neither predictor reached statistical significance. Contrary to previous studies, no associations were observed between deprivation, pharmacy workload, and pharmacy ownership^20^,^22^–^26^ and vaccination numbers.

Pharmacist interviews generally reflected the findings of previous research,^12^ and suggested that the relative performance of a pharmacy may be explained more adequately by the complex interplay between that pharmacy and external factors, specific to that pharmacy, than by population-level measures. The potential for the service to bring pharmacists and GPs into conflict is a key determinant of the approach taken by pharmacists. This is consistent with the findings of previous research,^23^,^25^,^35^ as are the findings suggesting that having a robust supply chain,^36^ and more than one pharmacist being available, facilitate service provision.^8^,^20^,^22^

### Implications for research and practice

These findings raise questions about how prepared community pharmacies are to take on services currently provided by GPs. The pharmacy profession has welcomed the availability of NHS vaccination from community pharmacies but the performance of individual pharmacies is variable and the overall contribution to vaccine uptake is low. Workload, lack of public awareness, vaccine procurement, health service disorganisation, and conflict with GPs were obstacles that prevented pharmacies making a greater contribution. Pharmacists, aware of the potential for conflict between themselves and GPs, moderated their behaviour to mitigate such risk. This has implications for both influenza vaccination and other services in which GPs and pharmacists may be seen to be competing for the same patients. The challenge for policymakers is to find a way of fostering collaboration between the professions for population benefit.

Interview findings suggest that, where there is insufficient capacity within community pharmacies to increase their range of services, they will prioritise service provision. In doing so the benefits of increased activity in one service will be offset by reductions in another. Prioritisation decisions will be made based on each pharmacist’s values, which may or may not be aligned with those of policymakers. It is conceivable that, in some cases, increasing the number of community pharmacy services could be counterproductive. Policymakers and community pharmacists must ensure that, in making them available, pharmacies have capacity to deliver existing as well as new services effectively.

Future research should explore the finding that pharmacies may be suited to vaccinating individuals who are aged <65 years and at risk (Table 2). Vaccination rates in this group remain persistently low. Promoting community pharmacies to this group may support efforts to increase uptake. Research is also warranted to quantify what effect having more than one pharmacist present at a pharmacy has on service delivery, and to verify whether pharmacists opting out of providing influenza services report similar barriers to service provision.

In the UK, increasing the range of services, including influenza vaccination, provided by community pharmacies is promoted as a means to reducing GP workload. For this to happen, obstacles including health service disorganisation, increasing pharmacist capacity, managing GP–pharmacy relationships, improving vaccine procurement arrangements both to mitigate the risk to GP practices of reduced service income and to remove disincentives for pharmacies, and finally, improving public awareness must be overcome. This requires action from pharmacists, GPs, and policymakers.

## Appendix 1. List of explanatory variables (predictors) used in analysis

**Table:**

**Predictor**	**Source**
Pharmacy ownership type	NHS Wales Shared Services Partnership
Rural Urban Classification	Office for National Statistics
WIMD	Public Health Wales Observatory
Pharmacy opening hours	NHS Wales Shared Services Partnership
Average monthly prescription volume (‘000s)	NHS Wales Shared Services Partnership

WIMD =Welsh Index of Multiple Depression.
